# Septic Arthritis of the Elbow: A 10-Year Retrospective Clinical and Microbiological Review From a Single Center

**DOI:** 10.7759/cureus.81044

**Published:** 2025-03-23

**Authors:** Omer Nasim, Khalid Saifullah Baig, Salman Khan, Aamir Khalil, Arsallan Karim, Mohammad Noah Khan, Mohammad Ahmed Arsalan Khan, Abdullah Durrani, Charalampos Pantelias

**Affiliations:** 1 Orthopaedics and Trauma, Salisbury District General Hospital, Southampton, GBR; 2 Rheumatology, St. Vincent's University Hospital, Dublin, IRL; 3 Surgery, Hayatabad Medical Complex Peshawar, Peshawar, PAK; 4 Trauma and Orthopaedics, Poole General Hospital, Poole, GBR; 5 Trauma and Orthopaedics, University Hospitals Dorset National Health Service (NHS) Foundation Trust, Poole, GBR; 6 Trauma and Orthopaedics, Royal Victoria Hospital, Belfast, GBR; 7 General Surgery, Tipperary University Hospital, Clonmel, IRL

**Keywords:** acute gout, calcium pyrophosphate dihydrate crystal deposition disease (cppd), crystal arthropathies, infectious arthritis, prevalence study, serum uric acid level

## Abstract

Introduction

In industrialized nations, the incidence of septic arthritis (SA) varies depending on geographic region, socioeconomic status, and age group. The condition is more frequently observed in male individuals, the elderly, and children. Additionally, its prevalence may have increased due to a rise in orthopaedic procedures, an aging population, and higher rates of immunosuppression. Hence, understanding the evolving clinical and epidemiological patterns of SA is crucial, along with identifying the common microbiological causes and pathogens involved.

Material (patients) and methods

A retrospective examination of the case series analysis was conducted. Patients underwent treatment at the Poole General Hospital, specializing in acute orthopaedic cases. Data collection lasted six months, from January 2021 to July 2021. During this time, every individual with a suspicion of primary SA of the elbow joint underwent aspiration. Exclusion criteria included any previous elbow joint surgery, a diagnosis of fungal or tuberculous arthritis, and duration of symptoms >6 months. Means and standard deviations were displayed for the continuous variables. Continuous data were summarized using averages and standard deviations, whilst categorical data was provided as absolute numbers with their corresponding percentages.

Results

A total of 241 patients were included in the study, comprising 200 (83%) male patients and 41 (17%) female patients, with a mean age of 67.47 ± 18.23 years. The most common symptom was pain (81.7%), while fever (>37.8°C) was observed in 3.7% of cases. The mean white blood cell (WBC) count was 11.85 ± 7.2, and C-reactive protein (CRP) levels averaged 103.45 ± 102. Leukocytosis was noted in 48.5% of cases. Comorbidities included hypertension (25.3%), diabetes (12.0%), and immunosuppression (34.0%). Gram stain identified bacteria in 10% of cases, while microbial cultures were positive in 27.1%, with *Staphylococcus aureus* being the most frequently isolated pathogen (58.8%). Resistance to co-trimoxazole was observed in 61.8% of isolates, with 79.4% being *Staphylococcus aureus* and 11.7% being methicillin-resistant *Staphylococcus aureus* (MRSA) (p<0.001). Univariate analysis showed significant associations between rheumatoid arthritis (RA), diabetes, and orthopaedic complications (p<0.01). Patients with clinical complications had a significantly longer hospital stay (17.8 ± 12.4 days vs. 6.43 ± 9.5 days, p<0.01). These findings highlight the importance of regional bacterial trends in guiding antibiotic therapy and patient management.

Conclusions

The findings emphasize the predominant role of *Staphylococcus aureus *as the causative pathogen, with notable antibiotic resistance patterns, particularly to co-trimoxazole and erythromycin. Despite timely surgical intervention and antibiotic therapy, complications including orthopaedic sequelae and prolonged hospital stays were more common among patients with RA, diabetes, and other comorbidities.

## Introduction

Septic arthritis (SA) is a quickly damaging joint ailment caused by microorganisms infiltrating the joint space, leading to the destruction of joints and loss of function. In western Europe, the incidence varies around 4-10 cases per 100,000 individuals annually. While it primarily affects weight-bearing joints in the lower limbs, upper limb joints can also be involved, albeit less frequently [1,2]. Elbow SA accounts for 6-9% of cases [3,4]. Western countries have witnessed an increasing trend in SA cases, influenced by factors like advanced age, comorbidities, skin infections, orthopaedic procedures, and underlying rheumatic disorders [2-5].

Furthermore, the prevalence is higher in lower socioeconomic populations, as evident in a northern European study [1]. In terms of gender distribution, SA predominantly affects male individuals at a higher frequency than female individuals, and children are more susceptible to SA than adults [6].

A high level of suspicion is necessary for SA in patients presenting with joint-related symptoms like swelling and pain, irrespective of age or medical status. However, there are some predisposing risk factors that put the patient at risk. These are rheumatoid arthritis (RA) and prosthetic joints, which elevate the occurrence of SA to 30-70 cases per 100,000, representing an almost tenfold increase in incidence. Mortality rates are higher in RA patients with septic joints due to joint damage and immunosuppressive medications. Diagnosis in these patients is challenging, often leading to delayed diagnosis as it can be mistaken for an RA flare. Corticosteroids, particularly intra-articular injections, increase the likelihood of SA [7]. Other joint diseases like osteoarthritis (OA), gout, and pseudogout predispose individuals to SA. Advanced age, diabetes mellitus, cancer, cirrhosis, chronic renal failure, which requires haemodialysis, alcoholism, joint prosthesis, and intravenous drug misuse are all additional risk factors. Skin infections also contribute to the risk of SA. Although monoarticular involvement is commonly observed, studies indicate that 10-16% of patients exhibit polyarticular involvement. Multiple microorganisms have been recognized as causing agents of SA [8-10].

However, it is crucial to consider that the causative microbe can only be identified through culture in nearly 65% of individuals. *Staphylococcus aureus* is the most common microbe in adults, comprising about 41-87% of cases [9-12]. The rise of methicillin-resistant *Staphylococcus aureus* (MRSA), especially in hospitalized patients and intravenous drug abusers, has also become a significant concern due to its association with severe infections, limited treatment options, prolonged hospital stays, and increased morbidity and mortality rates [13]. Beta-haemolytic streptococci are the second most common pathogens. Gram-negative organisms are more prevalent in elderly populations likely due to comorbidities such as urinary tract infections and cutaneous ulceration [14].

In sexually active adolescents and young adults, disseminated gonococcal infection (DGI) is the most prevalent type of bacterial arthritis, and it may also manifest in young children as a consequence of sexual abuse [15]. Gonococcal arthritis predominantly impacts the knee, with the elbow and ankle being the subsequent most commonly involved joints. Female individuals are disproportionately affected by gonococcal arthritis [16,17].

Infection can enter the joint through different routes: (a) hematogenous dissemination leading to pathogen lodging in synovial capillaries; (b) infection from nearby sources; and (c) direct inoculation through trauma or iatrogenic events like joint surgery or aspiration of the joint. A definitive entry point is determined in about 50% of all patients, with other typical routes including puncture wounds, surgical incisions, and contiguous infections [18]. The synovial tissue's vascularity and absence of a basal lamina facilitate the blood-borne transfer of bacteria into the joint. Once bacteria enter the joint space, a rapid inflammatory response occurs, leading to polymorphonuclear cell infiltration. These cells release enzymes that degrade the cartilage ground substance [19]. The interplay between the particular microorganism and the host's defense determines SA development. The inflammatory response can lead to irreversible loss of cartilage glycosaminoglycans despite prompt antibiotic treatment, resulting in joint destruction. Immunocompromised and hospitalized patients are at higher risk, particularly with invasive procedures, intravascular devices, or urinary catheters [5].

Diagnosis relies on clinical assessment, including patient history, physical examination, and investigations. Patients diagnosed with acute SA commonly exhibit symptoms such as pain, redness, swollen joints, tenderness, and limited movement of joints. The presentation of these symptoms typically spans one to two weeks and although their presence may not be consistent in all cases, a hallmark feature is the mild onset of fever, observed in about 30-40% of individuals, with only a fraction of these patients experiencing a temperature exceeding 39°C [5,20,21].

Blood test analyses reveal higher levels of white blood cell (WBC) count, C-reactive protein (CRP), and erythrocyte sedimentation rate (ESR) in individuals with SA. However, normal acute-phase reactants should not negate the possibility of SA. A recent study indicates that serum procalcitonin could potentially serve as a unique indicator of septic and non-septic arthritis, though its diagnostic precision requires further validation [1,11,22].

For a definitive diagnosis, aspiration of synovial fluid from the affected joint is essential. The collected synovial fluid samples undergo an examination to determine WBC count, leukocyte esterase level, alpha-defensin level, synovial CRP, and bacterial culture, which collectively aid in confirming the accurate diagnosis of SA. Gram staining identifies the causal agent in about 50% of cases, increasing to 67% with culture. However, it is advised that blood cultures must always be obtained before initiating antibiotic treatment to increase the likelihood of identifying the causative organisms. Imaging techniques such as CT scans and MRIs provide information on the degree of inflammation and tissue destruction but cannot reliably differentiate infectious from non­infectious etiologies of inflammatory arthritis [23,24]. However, MRI can assist in assessing coexisting osteomyelitis, which may indicate the need for orthopaedic intervention as well as evaluating deep joints (e.g., hip or sacroiliac joint).

SA can result in severe complications such as osteomyelitis and joint stiffness, with mortality rates ranging from 11.5-19% in older individuals. Early recognition, adequate drainage techniques, and antibiotic treatment are crucial for the risk reduction of joint injury and for preventing the progression of systemic complications. Delays in diagnosing and treating SA can result in irreversible joint damage, leading to functional impairment and even death [25,26].

This study aimed to delineate the epidemiological and clinical features of elbow SA patients who received treatment at a tertiary hospital over 10 years. It also aimed to explore the correlation between specific clinical and epidemiological factors and the occurrence of clinical or orthopaedic issues during treatment.

## Materials and methods

Study design

This research utilized a retrospective cohort study design, analyzing data from a continuous series of cases of native elbow SA. It aimed to investigate clinical and microbiological features as well as outcomes of patients who underwent diagnostic and treatment procedures over a defined 10-year period.

Study setting

The study was conducted in a single-center healthcare facility, with data collection derived from patients treated within the institution. Procedures including joint aspirations were performed in non-theatre settings such as the emergency department or hospital wards, following aseptic techniques.

Study population

The population included patients diagnosed with native elbow SA, characterized by clinical suspicion of infection in the elbow joint, who underwent joint aspiration and, when indicated, surgical intervention. The study period spanned from January 2012 to December 2021.

Sample size

The total sample size consisted of all eligible cases of native elbow SA identified within the study period. The exact number of cases depended on the retrospective review of records, which captured a continuous series of patients meeting the inclusion criteria.

Sampling technique

A purposive sampling method was employed, with data extracted for all cases that satisfied the inclusion criteria. This ensured that the study focused specifically on patients with the targeted diagnosis and excluded those with confounding conditions.

Inclusion criteria

Patients clinically suspected of having SA of the elbow joint and patients who underwent diagnostic joint aspiration and/or surgical intervention as part of their treatment were included.

Exclusion criteria

Patients with a history of metastatic disease affecting the elbow joint and patients under the age of 18 years were excluded.

Data collection

Data were obtained retrospectively from the institutional laboratory and patient records. Information was extracted into an Excel spreadsheet (Microsoft Corp., Redmond, United States) for analysis. The collected data included demographics (age, sex), clinical presentation and diagnostic findings, details of joint aspiration and microbiological results, treatment modalities including surgical interventions and outcomes, and follow-up data.

Data analysis

The dataset was systematically reviewed before analysis to ensure completeness and accuracy. Continuous variables were described using means and standard deviations, while categorical data were presented as absolute values alongside their respective percentages. Data normality was assessed to determine the appropriate statistical approach. Descriptive statistics were applied to summarize key trends within the dataset, offering an overview of patient demographics, clinical characteristics, and laboratory findings.

Quality assurance

Data accuracy and completeness were ensured through cross-checking with original patient records and laboratory databases. The use of standardized aseptic techniques for joint aspiration reduced procedural variability. The study adhered to established clinical and ethical guidelines, including the Declaration of Helsinki and good clinical practice protocols. To ensure validity, all data were reviewed by independent auditors from the clinical audit department. The diagnosis of SA was established by following Newman’s criteria [27]. The patients included in this study satisfied at least one of the following criteria: (i) separation of a pathogenic bacteria from an affected joint through synovial fluid culture; (ii) separation of a pathogenic bacteria from the blood through culture; or (iii) purulent turbid synovial fluid drained elbow joint in case of negative cultures from earlier usage of antibiotics.

The study also gathered a range of variables from the patients, including gender, age, clinical features, leukocyte count, serum CRP levels at the time of hospital admission, microscopy results, Gram staining results, history of earlier joint disease, synovial fluid culture and sensitivity outcomes, comorbidities, presence of immunosuppression, systemic and orthopaedic ailments, and duration of hospital stay.

Statistical analysis

P-values were calculated to assess the statistical significance of observed associations. The chi-squared test was used for categorical variables, while univariate analysis was conducted to explore potential predictors of clinical and orthopaedic complications. A significance threshold of 5% (p<0.05) was applied across all statistical tests. Analyses were performed using IBM SPSS Statistics v23.0 (IBM Corp., Armonk, United States).

Ethical statement

The data utilized in this study were obtained from the laboratory database and compiled into an Excel spreadsheet. No additional contact with patients occurred. This project was conducted as a service evaluation and did not require formal ethical approval. It was registered with the Poole General Hospital clinical audit department under approval #5372 and adhered to the guidelines of the Declaration of Helsinki and good clinical practice.

## Results

**Figure 1:**
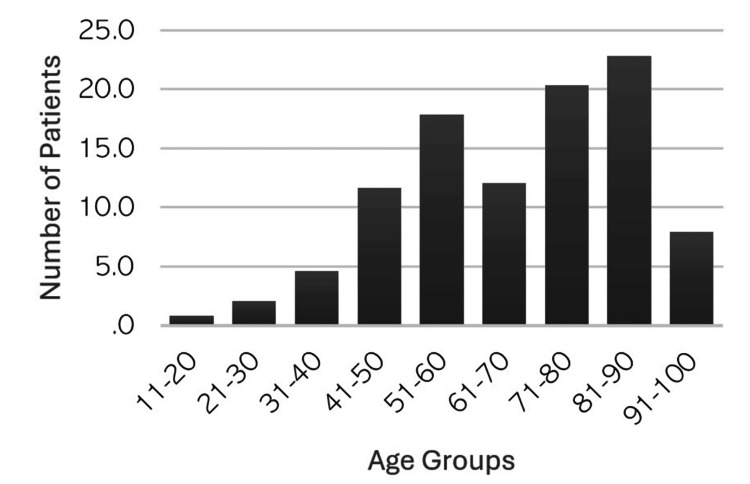
Distribution of elbow septic arthritis across age groups

A total of 241 patients were included, comprising 200 (83%) male patients and 41 (17%) female patients. The mean age of the participants was 67.47 ± 18.23 years. Figure 1 illustrates the distribution of patients across different age groups. Among the patients, 197 (81.7%) experienced pain, while nine (3.7%) exhibited fever with a body temperature above 37.8°C.

In the context of laboratory assessments, the mean WBC count was 11.85 ± 7.2, while the mean CRP level was 103.45 ± 102. Leukocytosis, defined as a leukocyte count exceeding 11,000, was identified in 96 (48.5%) cases. Additionally, 169 (86.2%) patients exhibited markedly elevated CRP levels (CRP >10 mg/dL).

Among the patients, 49 (20.3%) had a prior joint disease history. Additional analysis revealed that within this subgroup, 22 (9.1%) patients were diagnosed with RA, 23 (9.5%) with OA, and four (1.7%) with psoriatic arthritis (PsA).

Comorbidities were observed in the cohort, with 61 (25.3%) patients having hypertension and 29 (12.0%) patients having diabetes. Systemic diseases were present in 36 (14.9%) cases. A total of 82 (34.0%) patients were immunocompromised, with the most common cause being the use of corticosteroids or other immunosuppressants, observed in 46 (19.1%) cases. Among the immunocompromised patients, 24 (10%) had chronic renal failure (Table 1).

**Table 1:** Epidemiological and clinical aspects of joint aspiration in septic arthritis cases WBC: White blood cell; CRP: C-reactive protein; RA: Rheumatoid arthritis; PsA: Psoriatic arthritis; OA: Osteoarthritis

Parameter	Value
Gender	
Male	200 (83%)
Female	41 (17%)
Age	67.47 ± 18.23
Clinical features	
Pain	197 (81.7%)
Fever	9 (3.7%)
Laboratory levels	
Mean WBC count	11.85 ± 7.2
Leukocytosis	96 (48.5%)
CRP	103.45 ± 102.0
Marked elevated CRP	169 (86.2%)
Crystals on microscopy	
None	188 (78%)
Monosodium urate	32 (13.3%)
Calcium pyrophosphate	21 (8.7%)
Preexisting joint disease	49 (20.3%)
RA	22 (9.1%)
OA	23 (9.5%)
PsA	4 (1.7%)
Immune status	82 (34%)
Steroids or immunosuppressants used	46 (19.1%)
Chronic kidney disease	24 (10%)
Malignancies	10 (4.1%)
AIDS	1 (0.4%)
Hepatic cirrhosis	1 (0.4%)
Comorbidities	
Systemic hypertension	61 (25.3%)
Diabetes	29 (12.0%)
Systemic diseases	36 (14.9%)
Other	68 (28.2%)
Clinical complication	15 (6.2%)
Death	6 (2.5%)
Septic shock	1 (0.4%)
Acute myocardial infarction	2 (0.8%)
Pulmonary complication	3 (1.2%)
Acute kidney injury	3 (1.2%)
Orthopaedic complication	27 (11.2%)
Osteoarthrosis	7 (2.9%)
Rigidity	14 (5.8%)
Chronic osteomyelitis	3 (1.2%)
Surgical wound complication	3 (1.2%)
Length of hospital stay	7.14 ± 10.08

Clinical complications were observed in 15 (6.2%) patients, including death in six (2.5%) patients, septic shock in one (0.4%) patient, acute myocardial infarction in two (0.8%) patients, pulmonary complications in three (1.2%) patients, and acute kidney injury in three (1.2%) patients. Orthopaedic complications were noted in 27 (11.2%) patients, including rigidity in 14 (5.8%) patients, osteoarthrosis in seven (2.9%) patients, chronic osteomyelitis in three patients, and surgical wound complications in three patients (Table 1).

All patients underwent aseptic drainage of the elbow joint. A broad-spectrum antibiotic regimen was administered to all patients until the intraoperative culture results were available. Following the identification of specific bacteria from the cultures, patients received targeted antibiotic treatment tailored to the identified pathogens.

Microscopic investigation of synovial fluid samples for crystal identification yielded the following results: 188 (78%) cases showed no crystals; sodium urate crystals were identified in 32 (13.3%) patients; and calcium pyrophosphate crystals were detected in 21 (8.7%) cases. Gram staining of the synovial fluid revealed bacteria in 24 (10%) cases. However, microbial cultures were positive in 64 (27.1%) cases, with gram-positive bacteria identified in 60 (25.3%) patients and gram-negative bacteria in four (1.7%) patients. The discrepancy between Gram stain and culture results was observed in 40 (58.8%) patients, with a Gram stain sensitivity in 33 (13.54%) patients.

The most commonly isolated organism was *Staphylococcus aureus* detected in 40 (58.8%) patients. Group A *Streptococcus *and group B *Streptococcus *were identified in five (7.4%) and six (8.8%) patients, respectively.* Staphylococcus epidermidis* was found in three (4.4%) patients, and mixed coagulase-negative staphylococci were present in six (8.8%) culture results (Table 2).

**Table 2:** Distribution of Gram stain results and bacterial isolates in synovial fluid samples MRSA: Methicillin-resistant *Staphylococcus aureus*

Gram stain/organism	n (%)
Gram stain	
Gram-positive	24 (10%)
No organism seen	216 (90%)
Organisms isolated	68 (27.1%)
Bacterial isolates	
Staphylococcus aureus	40 (58.8%)
Group A *Streptococcus*	5 (7.4%)
Group B *Streptococcus*	6 (8.8%)
Staphylococcus epidermidis	3 (4.4%)
Mixed coagulase-negative staphylococci	6 (8.8%)
MRSA	4 (5.9%)
Pseudomonas aeruginosa	4 (5.9%)
Total	241 (100%)

Microbial antibiotic resistance analysis revealed that among the strains tested, 34 (61.8%) were resistant to co-trimoxazole and 15 (24.2%) were resistant to erythromycin. Among these co-trimoxazole-resistant strains, 27 (79.4%) were identified as *Staphylococcus aureus*, while four (11.7%) were identified as MRSA (p<0.001) (Table 3).

**Table 3:** Antibiotic sensitivity and resistance patterns of bacterial isolates

Antibiotic	Sensitive, n (%)	Resistant, n (%)	Intermediate, n (%)	Total, n (%)
Flucloxacillin	5 (55.6%)	4 (44.4%)	-	9 (100%)
Erythromycin	46 (74.2%)	15 (24.2%)	1 (1.6%)	62 (100%)
Clindamycin	38 (76.0%)	5 (10.0%)	7 (14.0%)	50 (100%)
Doxycycline	1 (50.0%)	1 (50.0%)	-	2 (100%)
Ciprofloxacin	9 (56.2%)	7 (43.8%)	-	16 (100%)
Co-trimoxazole	21 (38.2%)	34 (61.8%)	-	55 (100%)
Penicillin	8 (100%)	-	-	8 (100%)
Oxacillin	6 (85.7%)	1 (14.3%)	-	7 (100%)
Ceftazidime	34 (100%)	-	-	34 (100%)
Meropenem	3 (100%)	-	-	3 (100%)
Piperacillin-tazobactam	1 (100%)	-	-	1 (100%)
Ampicillin	1 (33.3%)	2 (66.7%)	-	3 (100%)
Cefuroxime	8 (72.7%)	3 (27.3%)	-	11 (100%)
Co-amoxiclav	42 (85.7%)	7 (14.3%)	-	49 (100%)
Tetracycline	8 (100%)	-	-	8 (100%)
Moxifloxacin	47 (94.0%)	3 (6.0%)	-	50 (100%)
Clarithromycin	47 (92.2%)	4 (7.8%)	-	51 (100%)
Rifampicin	41 (93.2%)	3 (6.8%)	-	44 (100%)
Gentamycin	43 (91.5%)	4 (8.5%)	-	47 (100%)
Total	368 (78.5%)	93 (19.8%)	8 (1.7%)	469 (100%)

The univariate analysis identified key associations between patient characteristics and the incidence of orthopaedic and clinical adverse events. Individuals with RA or diabetes had a markedly higher incidence of orthopaedic complications (p<0.01) (Table 4). Additionally, patients with clinical complications had an average hospital stay of 17.8 ± 12.4 days, which was significantly longer than the 6.43 ± 9.5 days observed in patients without complications (p<0.01). The existence of any comorbidities, particularly malignancies, was also strongly associated with the development of clinical complications (p<0.01) (Table 4).

**Table 4:** Key patient prognostic indicators for clinical and orthopaedic complications CRP: C-reactive protein; RA: Rheumatoid arthritis; PsA: Psoriatic arthritis; OA: Osteoarthritis; MRSA: Methicillin-resistant *Staphylococcus aureus*

Parameter	Clinical complication		P value	Orthopaedic complication		P value
	Yes	No		Yes	No	
Age (mean ± SD)	75 ± 12.8	66.9 ± 18.45	0.09	64.2 ± 17.4	68 ± 18.35	0.2
Gender						
Male	13 (5.4%)	187 (77.6%)	0.51	27 (11.2%)	173 (71.8%)	0.35
Female	2 (0.8%)	39 (16.2%)		7 (2.9%)	34 (14.1%)	
Clinical features						
Pain	14 (6.8%)	183 (88.8%)	0.50	30 (14.6%)	167 (81.1%)	0.59
Fever	1 (0.5%)	8 (3.9%)		1 (0.5%)	8 (3.9%)	
Laboratory levels						
Leukocytosis	8 (4%)	97 (49.0%)	0.59	20 (10.1%)	85 (42.9%)	0.16
Marked elevated CRP	15 (7.7%)	154 (78.6%)	0.09	29 (14.8%)	140 (71.4%)	0.31
Crystals on microscopy						
None	9 (3.7%)	179 (74.3%)	0.17	30 (12.4%)	158 (65.6%)	0.26
Monosodium urate	3 (1.2%)	29 (12%)		3 (1.2%)	29 (12%)	
Calcium pyrophosphate	3 (1.25)	28 (7.5%)		1 (0.4%)	20 (8.3%)	
Preexisting joint disease						
RA	1 (0.4%)	21 (8.7%)	0.59	9 (3.7%)	13 (5.4%)	<0.01
OA	0	23 (9.5%)	0.20	2 (0.8%)	21 (8.7%)	0.33
PsA	0	4 (1.7%)	0.77	1 (0.4%)	3 (1.2%)	0.45
Immune status						
Steroids or immunosuppressants used	2 (0.8%)	44 (18.4%)	0.42	10 (4.2%)	36 (15.1%)	0.07
Chronic kidney disease	0	24 (10%)	0.19	4 (1.7%)	20 (8.4%)	0.43
Neoplasms	4 (1.7%)	6 (2.5%)	<0.01	3 (1.3%)	7 (2.9%)	0.14
AIDS	0	1 (0.4%)	0.93	0	1 (0.4%)	0.86
Hepatic cirrhosis	0	1 (0.4%)	0.93	0	1 (0.4%)	0.86
Comorbidities						
Systemic hypertension	3 (1.2%)	56 (23.2%)	0.47	6 (2.5%)	53 (22%)	0.22
Diabetes	4 (1.7%)	30 (12.4%)	0.14	10 (4.1%)	24 (10%)	<0.01
Systemic diseases	2 (0.8%)	34 (14.1%)	0.60	6 (2.5%)	30 (12.4%)	0.39
Other	4 (1.7%)	63 (26.1%)	0.59	7 (2.9%)	60 (24.9%)	0.21
Clinical complication						
Death	0	0	0	2 (0.8%)	4 (1.7%)	0.21
Septic shock	0	0	0	1 (0.4%)	0	0.11
Acute myocardial infarction	0	0	0	0	2 (0.8%)	0.73
Pulmonary complication	0	0	0	1 (0.4%)	2 (0.8%)	0.36
Acute kidney injury	0	0	0	0	3 (1.2%)	0.63
Orthopaedic complication						
Osteoarthrosis	1 (0.4%)	6 (2.5%)	0.36	0	0	0
Rigidity	0	14 (5.8%)	0.39	0	0	0
Chronic osteomyelitis	3 (1.2%)	7 (2.9%)	<0.01	0	0	0
Surgical wound complication	0	3 (1.2%)	0.84	0	0	0
Days in hospital (mean ± SD)	17.8 ± 12.4	6.43 ± 9.5	<0.01	10.18 ± 12.8	6.6 ± 9.5	0.58
Bacteria isolated						
Staphylococcus aureus	4 (1.7%)	36 (14.9%)	0.22	7 (2.9%)	33 (13.7%)	0.32
Group A *Streptococcus*	0	5 (2.1%)	0.72	1 (0.4%)	4 (1.7%)	0.53
Group B *Streptococcus*	0	6 (2.5)	0.67	0	6 (2.5%)	0.39
Staphylococcus epidermidis	0	3 (1.2%)	0.84	1 (0.4%)	2 (0.8%)	0.36
Mixed coagulase negative	1 (0.4)	5 (2.1%)	0.32	2 (0.8%)	4 (1.7%)	0.20
MRSA	0	4 (1.7%)	0.77	0	4 (1.7%)	0.54
Pseudomonas aeruginosa	0	5 (1.7%)	0.77	2 (0.8%)	2 (0.8%)	0.09

Additionally, the univariate statistical analysis revealed significant correlations between the use of steroids and immunosuppressants and the culture of bacteria from patient synovial fluid (p=0.04). Moreover, patients diagnosed with RA (p=0.02) and diabetes (p=0.05) exhibited significant correlations with bacterial isolation. The mean duration of hospital stay for patients with positive bacterial culture was significantly higher at 9.9 ± 12.3 days, compared to 6.0 ± 8.8 days in those without bacterial culture positivity (p<0.01) (Table 5).

**Table 5:** Comparative analysis of bacterial isolates and clinical features CRP: C-reactive protein; RA: Rheumatoid arthritis; PsA: Psoriatic arthritis; OA: Osteoarthritis

Parameter	Bacteria isolated		P value
	Yes	No	
Age (mean ± SD)	65 ± 18.3	68 ± 18.1	0.24
Gender			
Male	60 (24.9%)	140 (58.1%)	0.12
Female	8 (3.3%)	33 (13.7%)	
Clinical features			
Pain	14 (6.8%)	183 (88.8%)	0.50
Fever	1 (0.5%)	8 (3.9%)	
Laboratory levels			
Leukocytosis	8 (4%)	97 (49.0%)	0.59
Marked elevated CRP	15 (7.7%)	154 (78.6%)	0.09
Crystals on microscopy			
None	62 (25.7%)	126 (52.3%)	0.34
Monosodium urate	5 (2.1%)	27 (11.2%)	
Calcium pyrophosphate	1 (0.4%)	20 (8.3%)	
Previous joint disease			
RA	11 (4.6%)	11 (4.6%)	0.02
OA	7 (2.9%)	16 (6.6%)	0.48
PsA	1 (0.04%)	3 (1.2%)	0.68
Immune status			
Steroids or immunosuppressants used	18 (7.5%)	28 (11.7%)	0.04
Chronic kidney disease	4 (1.7%)	20 (8.4%)	0.14
Neoplasms	3 (1.3%)	7 (2.9%)	0.56
AIDS	0	1 (0.4%)	0.72
Hepatic cirrhosis	0	1 (0.4%)	0.72
Comorbidities			
Systemic hypertension	11 (5.8%)	45 (18.7%)	0.23
Diabetes	14 (5.8%)	20 (8.3%)	0.05
Systemic diseases	2 (0.8%)	34 (14.1%)	0.60
Other	15 (6.2%)	52 (21.6%)	0.13
Clinical complication			
Death	3 (1.2%)	3 (1.2%)	0.22
Septic shock	1 (0.4%)	0	0.28
Acute myocardial infarction	0	2 (0.8%)	0.51
Pulmonary complication	0	3 (1.2%)	0.36
Acute kidney injury	1 (0.4%)	2 (0.8%)	0.63
Orthopaedic complication			
Osteoarthrosis	0	7 (2.9%)	0.09
Rigidity	3 (1.2%)	11 (4.6%)	0.40
Chronic osteomyelitis	9 (3.7%)	1 (0.4%)	<0.01
Surgical wound complication	1 (0.4%)	2 (0.8%)	0.63
Days in hospital (mean ± SD)	9.9 ± 12.3	6 ± 8.8	<0.01
Gram stain			
Gram-positive	24 (10%)	0	0.00

## Discussion

This research provides a demographic and clinical assessment of 241 cases, with a median age of 67 years. This age distribution aligns with previous studies reporting a mean age of above 60 years. Among the sample, the immunocompromised patients were 82 (34%), 50 (20%) had previous joint disease, and 189 (79%) had other comorbidities. Earlier studies have identified a link between SA and immune system dysfunction [28-30], various comorbidities [6,30], along with prior joint diseases, including OA and RA [14].

In this research, 22 (9.1%) individuals had RA, which is comparable to a previous study reporting a prevalence of RA in 17 (7%) patients with SA, although that study also included cases relating to the elbow and shoulder joints [31]. Furthermore, this study revealed correlations between patients diagnosed with RA, diabetes, and the use of steroids and other immunosuppressants with bacterial isolation through culture. These findings suggest that the aforementioned conditions increase the likelihood of SA. Previous literature has also examined these factors, identifying RA as a significant risk factor for SA. Estimates suggest that patients with RA have a four to eight-time rise in the prevalence of SA in comparable European residents [1,3,32].

Furthermore, a marked elevation of CRP (CRP >10 mg/dL) was seen in 169 (86.2%) cases, a result comparable to another study's findings where elevated CRP was seen in 95% of the participants. Leukocytosis was detected in 96 (48.5%) cases, which is higher than the findings of one study [14] that reported 77 (42%), but it falls within the range of 50% to 68% as reported by other authors [14]. The mean CRP level in this study was 103.45, which was comparable to another study that reported a mean CRP level of 107 mg/dL [33]. Regarding synovial fluid microscopy, the current study found that 32 (13.3%) patients had sodium urate crystals, while 21 (8.7%) patients had calcium pyrophosphate crystals. However, another study found that 4% of individuals with SA had monosodium urate crystals and 7.3% had calcium pyrophosphate crystals [34]. Among the patients in this study, the causative agent was diagnosed by synovial fluid culture resulting in 64 (27.1%) cases. Similarly, other authors have stated detection rates fluctuate from 82% to 95% for the responsible bacteria [3,14,28,29,33].

In this study, Gram staining only detected microorganisms in 24 (10%) cases, resulting in a sensitivity of 33 (13.5%), which is significantly lower than the sensitivity of 22% reported in another investigation [35]. *Staphylococcus aureus *was the major pathogen identified in this cohort, growing in 139 (58%) of the positive synovial fluid cultures. This microbial profile aligns with existing literature, wherein* S. aureus *accounts for 42% to 77% of infections [36]. The antimicrobial sensitivity conducted in this research indicated that 34 (61.8%) showed resistance to co-trimoxazole and 15 (24.2%) demonstrated resistance to erythromycin. Of the co-trimoxazole-resistant strains, the majority, 27 (79.4%), were identified as *S. aureus*, including four (11.7%) that were MRSA strains. Another study reported a 36.9% resistance rate for co-trimoxazole and a 48.8% resistance rate for erythromycin in staphylococcal isolates, but it focused on a different population (Yongor population aged 1-5 years) without considering the specific type of infection. In this study, 26 (11%) patients had orthopaedic complications during follow-up, mainly joint stiffness in 13 (5.8%) patients and chronic osteomyelitis in seven (2.9%) patients [37]. These rates were lower than those reported by Weston et al. [14], where 15% of patients experienced elbow stiffness and 15% developed chronic osteomyelitis subsequent treatment for elbow SA. Diabetes and RA as a preexisting joint disease exhibited a significantly higher rate of orthopaedic complications in these patients [31].

Although specific data on how age or comorbidities influence orthopaedic complications in SA were not found in the literature, several studies have highlighted important risk factors. These include immunosuppression, the use of immunosuppressive medications, and conditions like diabetes [9,38]. In this study, inpatient clinical complications were seen in 15 (6.2%) patients, with mortality being the most common, affecting six (2.6%) patients. Additional comorbidities included acute kidney injury and pulmonary issues, each occurring in three (1.2%) cases. The hospital stay increased to nearly three times longer in patients who developed complications than those who did not develop any complications. The recorded mortality rate of six (2.6%) was marginally lower than the 6-11.5% range documented in studies on SA affecting multiple joints [3,14] but higher than the 5-17% mortality range reported in studies on shoulder SA [28,34].

This study primarily examined individuals who had surgical drainage for SA, excluding patients who were managed with antibiotics alone, potentially leading to selection bias. The analysis relied on a univariate approach, assessing singular prognostic factors associated with adverse events in SA. A multivariate analysis, which could account for and evaluate multiple prognostic variables simultaneously, would have reduced the impact of confounding factors. However, the sample size was insufficient to support such an analysis.

The limitations of this study include its retrospective design, which restricted the ability to incorporate new parameters, relying solely on the data available for subsequent analysis. Additionally, the study was conducted at a single center, which, despite the significance of the extended data collection period, limits the generalizability of the findings to larger populations. The results are primarily applicable to the regional antibiotic policy of the specific location.

## Conclusions

This study provides a comprehensive demographic, clinical, and microbiological analysis of SA of the elbow, reinforcing known risk factors such as advanced age, immunosuppression, and preexisting joint disease. The findings highlight the predominant role of *Staphylococcus aureus *as the causative pathogen, with notable antibiotic resistance patterns, particularly to co-trimoxazole and erythromycin. Despite timely surgical intervention and antibiotic therapy, complications including orthopaedic sequelae and prolonged hospital stays were more common among patients with RA, diabetes, and other comorbidities. 

The study’s limitations, including its retrospective nature and single-center design, should be considered when interpreting the results. Nonetheless, the findings emphasize the importance of early diagnosis, targeted antimicrobial therapy, and vigilant management of high-risk patients to improve clinical outcomes. Future research incorporating larger, multicenter cohorts and multivariate analysis could further refine risk stratification and optimize treatment strategies for RA of the elbow.
